# Whistle while you work: improving psychiatry training in a London NHS Trust and what we learned along the way

**DOI:** 10.1192/bjo.2021.383

**Published:** 2021-06-18

**Authors:** Emily Duncan, Alex Bailey, Simon Edwards, Alison Butler, Layth Humsi

**Affiliations:** Central and Northwest London NHS Trust

## Abstract

**Aims:**

The aim of this project is to improve the training experience of Psychiatry trainees across CNWL. In QI terms, we want to achieve a satisfaction rating of above 7/10 for all themes identified by August 2021.

**Method:**

Collected baseline data on satisfaction and priority ratings on 7 training themes Held discussion groups with trainees for specific themes to generate issues and solutions Developed and provided Quality Improvement training for trainees and trainers, 1:1 support and QI clinics – empowering trainees to develop their own local project and to make changes to issues on the ground Enacted central changes in communication, responsiveness, recognising success.

Reassessed and fedback to the trainees throughout.

**Result:**

Our baseline satisfaction survey was completed in June 2020. Trainees their satisfaction for each theme out of 10 and to rank their priorities for change. Results showed satisfaction was lowest in morale and in safety and highest in education and supervision. Their highest priorities for change were safety, then morale, with induction as the lowest priority.

We repeated the survey in October 2020. This showed improvements in most themes (apart from induction, perhaps due to induction having to be delivered virtually). Satisfaction in key priority areas of morale and safety increased from 4.53 to 6.37, and 5.12 to 6.70 respectively. We also asked what ‘one thing’ would they improve about their training. Key phrases included teaching, on-call, communication and induction.

From this data, and softer feedback from trainees, it is encouraging that we are moving in a positive direction, but we are continuing to make changes.

**Conclusion:**

- Trainees must be central to the work in improving their training
- Using QI methodology helps – developing a structure and breaking down a bigger task helps make a plan
- Feedback is key – but people are busy and receive a lot of emails and requests to fill surveys – catching people ‘in person’ (virtually) was the best way to ensure a lot of responses
- Trainees have loads of great ideas, but they need support, time and resources to be able to develop their projects and changes
- Flexibility is crucial: some topics work better locally, driven by trainees and some require a more coordinated, central role

We hope that developing a structured approach to a large task like improving training will help make changes sustainable, and enables us to share our learning with others.

